# Supplementary material for: Causal interplay between lactose intolerance and gut microbiota: a combined bidirectional Mendelian randomization and in vivo validation study
Source: Front Nutr. 2026 Jun 1;13:1803337. doi: 10.3389/fnut.2026.1803337 (PMC13265576; doi:10.3389/fnut.2026.1803337)
Supplement: Supplementary file 1 [file Data_Sheet_1.zip › supplementary materials/Forward/leave one out cross validation/ebi-a-GCST90027641.finngen_R12_E4_LACTONAS.pdf]

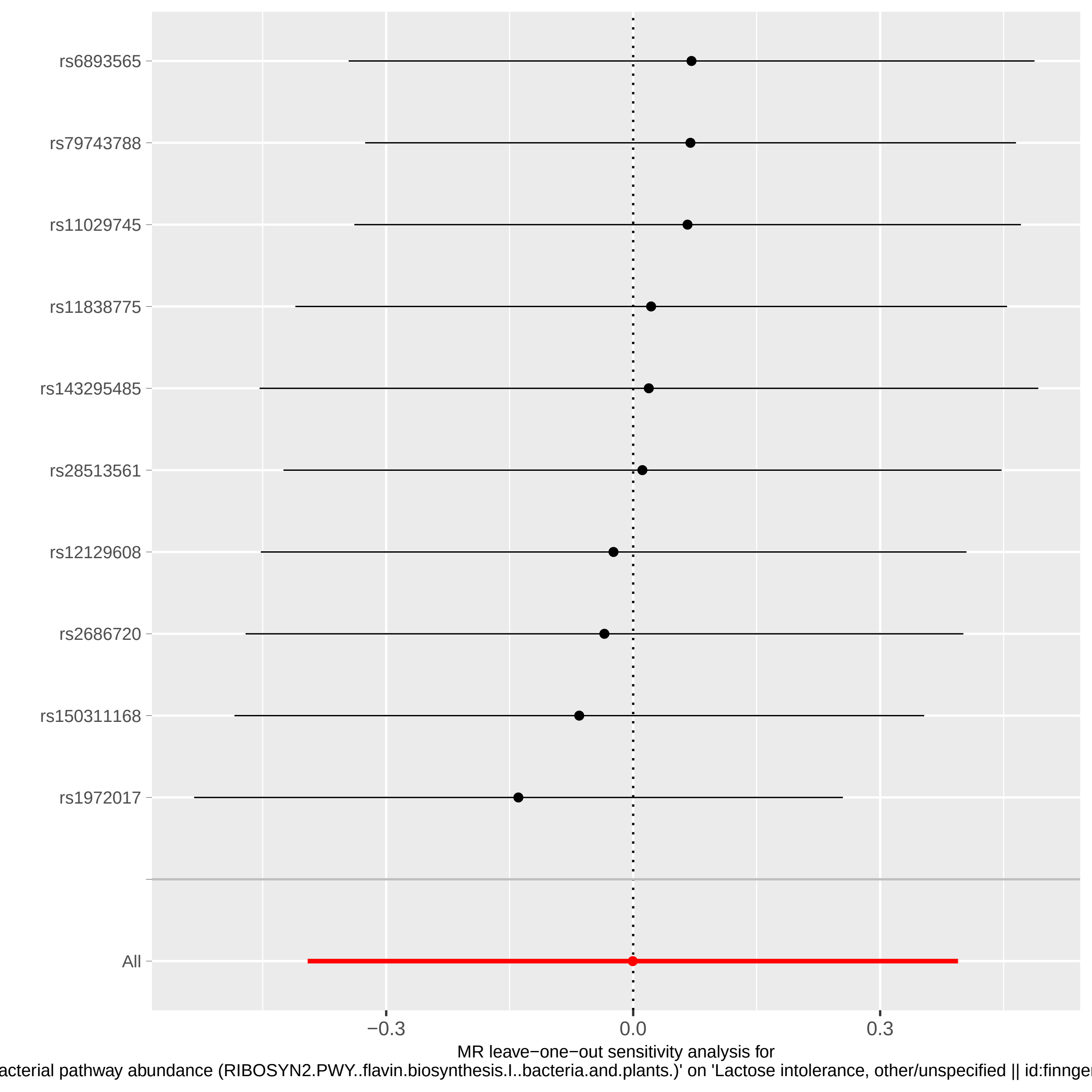
rs6893565
rs79743788
rs11029745
rs11838775
rs143295485
rs28513561
rs12129608
rs2686720
rs150311168
rs1972017
All
−0.3
0.0
0.3
MR leave−one−out sensitivity analysis for
acterial pathway abundance (RIBOSYN2.PWY..flavin.biosynthesis.I..bacteria.and.plants.)' on 'Lactose intolerance, other/unspecified || id:finnge
